# Evaluation method of Driver’s olfactory preferences: a machine learning model based on multimodal physiological signals

**DOI:** 10.3389/fbioe.2024.1433861

**Published:** 2024-12-18

**Authors:** Bangbei Tang, Mingxin Zhu, Zhian Hu, Yongfeng Ding, Shengnan Chen, Yan Li

**Affiliations:** ^1^ School of Intelligent Manufacturing Engineering, Chongqing University of Arts and Sciences, Chongqing, China; ^2^ Department of Physiology, Army Medical University, Chongqing, China; ^3^ School of Mechanical Engineering, Sichuan University of Science & Engineering, Yibin, Sichuan, China

**Keywords:** driving comfort, in-vehicle fragrance, olfactory preference, physiological signal, machine learning

## Abstract

**Introduction:**

Assessing the olfactory preferences of drivers can help improve the odor environment and enhance comfort during driving. However, the current evaluation methods have limited availability, including subjective evaluation, electroencephalogram, and behavioral action methods. Therefore, this study explores the potential of autonomic response signals for assessing the olfactory preferences.

**Methods:**

This paper develops a machine learning model that classifies the olfactory preferences of drivers based on physiological signals. The dataset used for training in this study comprises 132 olfactory preference samples collected from 33 drivers in real driving environments. The dataset includes features related to heart rate variability, electrodermal activity, and respiratory signals which are baseline processed to eliminate the effects of environmental and individual differences. Six types of machine learning models (Logistic Regression, Support Vector Machine, Decision Tree, Random Forest, K-Nearest Neighbors, and Naive Bayes) are trained and evaluated on this dataset.

**Results:**

The results demonstrate that all models can effectively classify driver olfactory preferences, and the decision tree model achieves the highest classification accuracy (88%) and F1-score (0.87). Additionally, compared with the dataset without baseline processing, the model’s accuracy increases by 3.50%, and the F1-score increases by 6.33% on the dataset after baseline processing.

**Conclusions:**

The combination of physiological signals and machine learning models can effectively classify drivers' olfactory preferences. Results of this study can provide a comprehensive understanding on the olfactory preferences of drivers, ultimately enhancing driving comfort.

## 1 Introduction

Driving comfort was a critical consideration in automotive design, prompting automakers to enhance driving comfort by using in-vehicle fragrances to improve the odor environment (Mustafa et al., 2016; Gentner et al., 2021). However, the current evaluation methods were unable to effectively assess the olfactory preferences of drivers, leading to a lack of sufficient consideration for driver olfactory preferences in the design process of in-vehicle fragrances. This resulted in existing in-vehicle fragrances struggling to achieve the expected effects of improving the odor environment and enhancing driving comfort. Therefore, it was crucial to find an effective olfactory preferences evaluation method to provide an important reference for in-vehicle fragrance designers and significantly enhance the driver’s comfort during driving.

The previously available evaluation methods could not effectively assess drivers’ olfactory preferences, indicating that the adopted approaches were not suitable for assessing olfactory preferences (Seok et al., 2017; Williams et al., 2023; Schacht et al., 2009). For example, in subjective evaluation methods, a large number of studies used rating scales to assess olfactory perception preferences (Yang W. et al., 2024; Martínez-Pascual et al., 2023), including evaluating the pleasantness of odors (Sorokowska et al., 2022; Farahani et al., 2023; Klyuchnikova et al., 2022) and the intensity of odors (Fjaeldstad et al., 2019; Lesur et al., 2023). However, these evaluation methods were highly subjective, and it often led to evaluation results being contrary to reality since drivers lacked the professional olfactory training (Hakyemez et al., 2013; Han et al., 2021; Seok et al., 2017). The use of electroencephalogram (EEG) methods could accurately determine the brain activity associated with drivers’ olfactory preferences (Nakahara et al., 2020; Islam et al., 2018). However, this method was difficult to apply (Abenna et al., 2023), demanding significant time and economic resources (Williams et al., 2023), which was the primary reason why electroencephalogram methods had not been widely adopted. Additionally, olfactory perception preferences could also be better evaluated through collecting behavioral data (Naudon et al., 2020; Ryan et al., 2008), but behavioral data could only reflect a part of physical indicators and could not fully reflect olfactory feelings. Actually, there was a correlation between physiological signals (such as electrodermal activity, heart rate, and respiratory signal) and olfactory preferences in the human body (Laureanti et al., 2022; Aurup, 2011). In the field of emotion research, physiological signals could characterize feelings of pleasure and disgust (Khare et al., 2023; Cai et al., 2023; Singh et al., 2023). Therefore, this provided a potential approach for assessing olfactory perceptual preferences. However, this finding had not yet been incorporated into olfactory preference evaluation methods.

The physiological signals of the human body were spontaneous responses used to assess physiological and psychological states (Li et al., 2023; Saha et al., 2024). Research had shown that the autonomic nervous system of the human body underwent changes after olfactory training, allowing for the identification of individual preferences for specific odors by observing different autonomic responses (Parreira et al., 2023). In addition, researchers had also found that experiencing different fragrances could alter skin-related physiological signals (Jiao et al., 2023). These results had potential practical value. However, single-modal physiological signals cannot comprehensively reflect an individual’s physiological changes and are easily influenced by external factors. Therefore, multimodal signals offer greater advantages in physiological monitoring (Garcia-Ceja et al., 2018; Debie et al., 2019).

To achieve a better recognition accuracy and a more stable recognition model, researchers explored physiological state recognition methods that integrated multimodal physiological signals. Chen et al. (2022) integrated electroencephalogram, heart rate variability, and electromyography signals into a convolutional neural network model, achieving alertness detection of drivers. Gong et al. (2024) designed a multimodal fusion method that simultaneously considered heterogeneity and correlation and explored the optimal combination of various physiological signals. Kose et al. (2021) used a fusion of horizontal eye movement, vertical eye movement, zygomatic muscle electrical activity, and trapezius muscle electrical activity to improve existing emotion recognition methods. Jia et al. (2022) proposed a domain adversarial learning squeeze and excitation network based on multimodal physiological signals to capture the characteristics of electroencephalogram (EEG) and electrooculogram (EOG) during sleep staging. The results showed that multimodal signals were effective for sleep staging tasks. For further research and convenience. This study chose to integrate electrodermal activity (EDA) (Shehu et al., 2023), heart rate variability (HRV) (Martis et al., 2014), and respiration (RESP) (Shin et al., 2022) features as indicators to assess drivers’ olfactory preferences.

This study aims to develop a machine learning model that using multiple physiological signals to evaluate drivers’ olfactory preferences. The ultimate goal is to assist automotive designers in creating in-vehicle fragrances that aligned with drivers’ olfactory preferences, enhancing driver comfort during driving. The contribution of this study could be summarized as the following:

(1) The experimental setting of this study is conducted in a real road environment, which allows the physiological response of drivers when experiencing in-vehicle fragrances to be closer to the real situation. To eliminate the influence of driving on physiological signals, a calm driving phase is implemented.
(2) To eliminate the influence of individual physiological differences, baseline processing is applied to collect physiological signal features. Additionally, to maxim-ize the retention of information from the original features, feature concatenation is employed for feature fusion.
(3) Six machine learning models are evaluated in this study. Results show that the decision tree model performed a better accuracy than the other models. In addition, the predicted results also show that models used in the work performed better in predicting the “disgust” preferences than that of the “like” preferences.

## 2 Materials and methods

**FIGURE 1:**
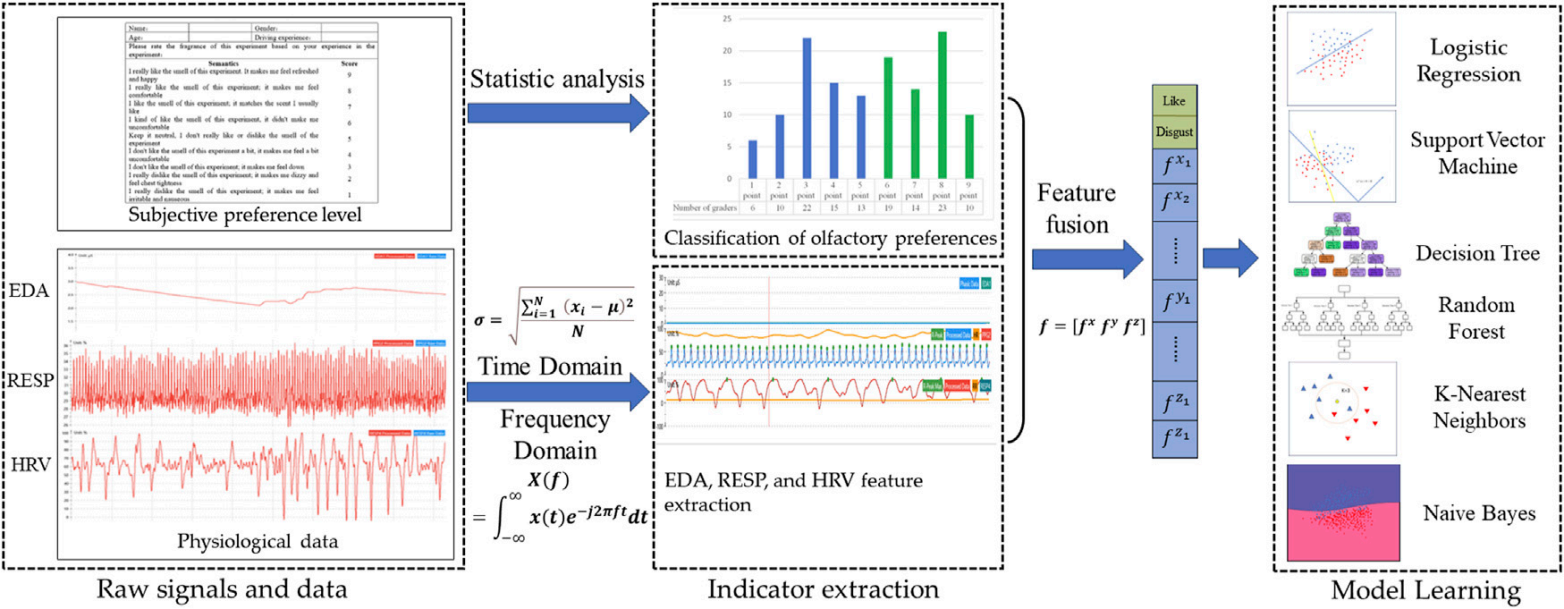
A framework for identifying driver olfactory preferences based on multiple physiological signals.”

The dataset used for training in this study comprises 132 olfactory preference samples collected from 33 drivers. To assess olfactory preferences, the participating drivers (Section 2.1) are strictly screened. This paper uses the ErgoLAB multichannel physiological instrument to collect physiological signals from these candidates and uses a 9-point hedonic scale to collect olfactory preference data from drivers (Section 2.2). In addition, the time domain and frequency domain analysis on the collected physiological data are conducted to obtain a feature set (Section 2.3). Finally, this study uses the driver’s olfactory preference data and the driver’s physiological signal feature set as the target label and the input feature, respectively. The performance of six machine learning models is trained (Section 2.4) and evaluated (Section 2.5) to predict the driver’s olfactory preference. The identification framework of drivers’ olfactory preferences is shown in Figure 1.

### 2.1 Participant

This study selects 33 drivers (16 males and 17 females) as participants through three rounds of screening. All participants are between 21 and 32 years of age (mean = 26.7 years; standard deviation = 2.6 years), with an average driving experience of 3.7 years (standard deviation = 2.1; range = 8–1 years). The experimental content and procedures of this experiment are approved by the Ethics Committee of Chongqing University of Arts and Sciences (approval no. CQWL202403). Firstly, the first round of screening eliminates drivers with central nervous system diseases and rhinitis. Secondly, subjective judgment tests are conducted in the second round of screening to ensure that participants can accurately judge their preferences. Finally, the third round of screening eliminates participants whose vital signs such as heart rate and breathing are not within the normal range before the experiment, as well as participants who have eaten heavy flavored foods before the experiment. After obtaining the participant’s consent, an informed consent form is signed with the participants, and they are informed on the experiment content and tasks to be completed during the experiment.

### 2.2 Equipment and procedure

**FIGURE 2:**
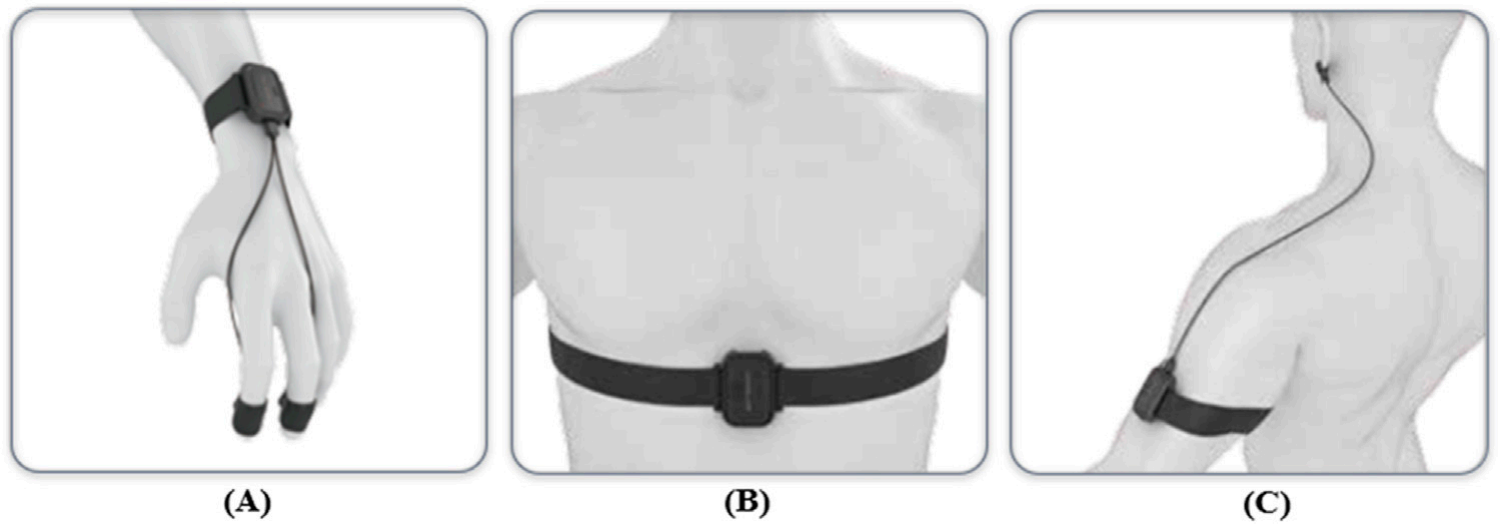
The wearing diagram of physiological signal sensor. **(A)** The wearing Diagram of EDA sensor; **(B)** The wearing Diagram of RESP sensor; **(C)** The wearing Diagram of PPG sensor.

In this study, peppermint essential oil, jasmine essential oil, sweet orange essential oil, and lavender essential oil are used as odor sources (the essential oil reagents are obtained from Refined Aroma and are non-toxic and harmless to humans). These odors are widely used in experiments and life (El Hachlafi et al., 2023; Karimi et al., 2024; Diass et al., 2021). The generation of odors is achieved through olfactory testing experience instruments (Tang et al., 2019; Tang et al., 2022). The ErgoLAB signal acquisition module is used to collect and record the physiological signals of participants (Zheng et al., 2021; Qu and Xie, 2023). The program for the experimental process is written through the ErgoLAB human-computer interaction platform (Liu et al., 2019). Specifications of the physiological signal data acquisition equipment are as follows:

(1) ErgoLAB EDA wireless electrodermal sensor (sampling rate: 64 Hz, acquisition range: 0–30 μS). The two electrodes of the EDA sensor are fixed at the fingertips of the index finger and middle finger (as shown in Figure 2A).
(2) ErgoLAB RESP wireless respiratory sensor (sampling rate: 64 Hz; acquisition range: 0–140 rpm). The belt of the RESP sensor is fixed between the chest and abdomen of the subject (as shown in Figure 2B).
(3) ErgoLAB PPG wireless blood volume pulse sensor (sampling rate: 64 Hz; acquisition range: 0–240 bpm). The ear clip electrodes of the PPG sensor are fixed on the earlobe (as shown in Figure 2C).

In addition, this paper obtains the olfactory preference data of drivers through a 9-point hedonic scale (Xia et al., 2020). The 9-point hedonic scale is used to evaluate “like”, but it is often used to measure preferences (Barnett et al., 2019; English et al., 2019). It consists of nine different categories of semantic components, ranging from ‘extreme dislike’ to ‘extreme like’.

**FIGURE 3:**
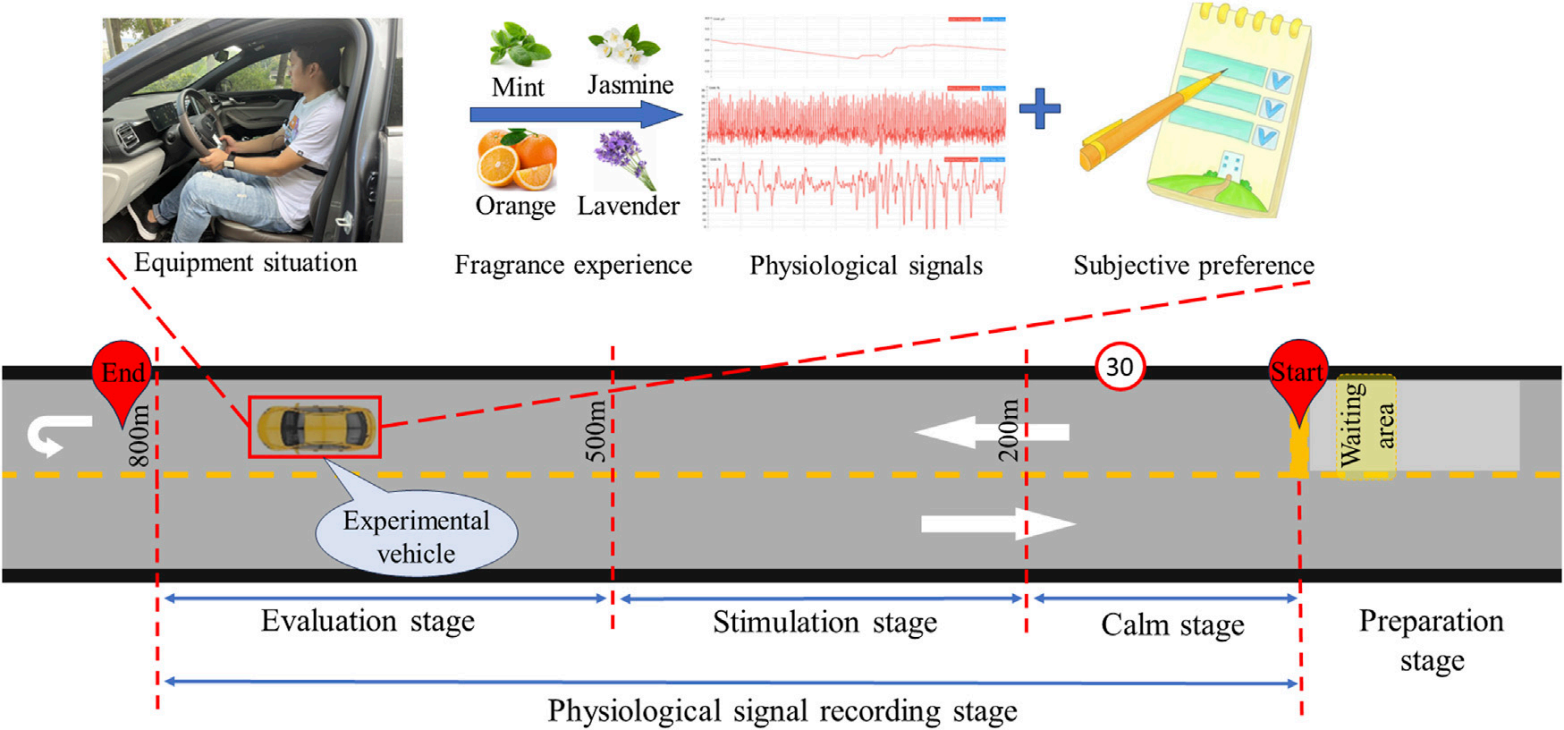
The experimental process and schematic diagram of experimental scenarios.

The experimental scenario is a closed two-lane highway, which is a straight road (speed limit of 30 km/h). The starting point and ending point of the experiment are about 800 m apart. The experimental vehicle is an automatic transmission vehicle without any odor. The experimental road is divided into four areas (preparation stage, calm stage, stimulation stage, and evaluation stage). The area 200 m forward from the starting point is the calm stage, the area 300 m forward from the end of the calm stage is the fragrance stimulation stage, and the area 300 m forward from the end of the stimulation stage is the olfactory preference collection stage. This experimental scenario is diagramed in Figure 3.

Each driver needs to complete four rounds of driving tasks, which means completing the experiments in the mint group, jasmine group, sweet orange group, and lavender group. The four groups of experiments are conducted randomly. The driving task for each lap includes four stages (preparation stage, calm stage, stimulation stage, and evaluation stage). Before each round of the experiment, the participants need to complete the preparation stage tasks in the waiting area. During the preparation stage, the experimenter needs to deodorize the car to ensure that there is no odor affecting the subsequent experiment. The participant needs to drive the vehicle and maintain a stable state of mind in the calm stage. When the vehicle enters the stimulating stage area, the fragrance begins to be released. After completing all the experimental tasks, the vehicle turns around and return to the waiting area to repeat the above experimental tasks. The scoring table was completed during the evaluation stage (as shown in Table 1).

**TABLE 1:** The 9-point hedonic scale.

The semantics of preferences	The value of preference
I extremely like the odor of this experiment	9
I like the odor of this experiment very much	8
I moderately like the odor of this experiment	7
I slightly like the odor of this experiment	6
I neither like nor dislike the odor of this experiment	5
I slightly dislike the odor of this experiment	4
I moderately dislike the odor of this experiment	3
I dislike the odor of this experiment very much	2
I extremely dislike the odor of this experiment	1

### 2.3 Feature extraction

Feature extraction refers to extracting representative and distinguishable features from raw data to describe the attributes and characteristics of the data. Feature extraction plays a crucial role in machine learning (Escobar-Linero et al., 2022). This study extracts electrodermal activity (EDA), heart rate variability (HRV), and respiratory signal-related features from 132 olfactory preference samples as the basis for evaluating olfactory preference. Table 2 shows the features extracted from three physiological signals.

**TABLE 2:** The physiological signal features extracted from heart rate variability (HRV), electrodermal activity (EDA), and respiratory signals (RESP), along with their corresponding symbols.

Psychophysiology signals	Feature extraction method	Characteristic symbol	Number of features
HRV	Time domain	HR, SDNN, RMSSD, SDSD, SD1, SD2	6
	Frequency domain	HF, LF	2
RESP	Time domain	RESP_Mean, Std	2
	Frequency domain	Power	1
EDA	Time domain	SC_Mean, SC_Max, SC_Min, Std	4

In addition, this paper uses changes in physiological signals as the input features of the model, meaning that all the input features of the model undergo baseline processing (Maithri et al., 2022). Specifically, the final feature is the difference of the physiological signal feature values extracted during the stimulation phase and the calm phase (Feature = Feature_Stimulation_ − Feature_Calm_).

#### 2.3.1 Time domain feature extraction

In time domain analysis, this paper calculates the mean (M), standard deviation (SD), and range-related features of three physiological signals. Due to the importance of R-wave detection in the time domain analysis of HRV (Gupta et al., 2020), the following extracting features are focused: standard deviation of normal and normal intervals (SDNN), root mean square of successive differences in adjacent intervals (RMSSD), standard deviation of successive differences in adjacent intervals (SDSD), standard deviation of instantaneous heartbeat interval *R-R* variability (SD1), standard deviation of continuous long-term *R-R* variability (SD2), and heart rate (HR).

**FIGURE 4:**
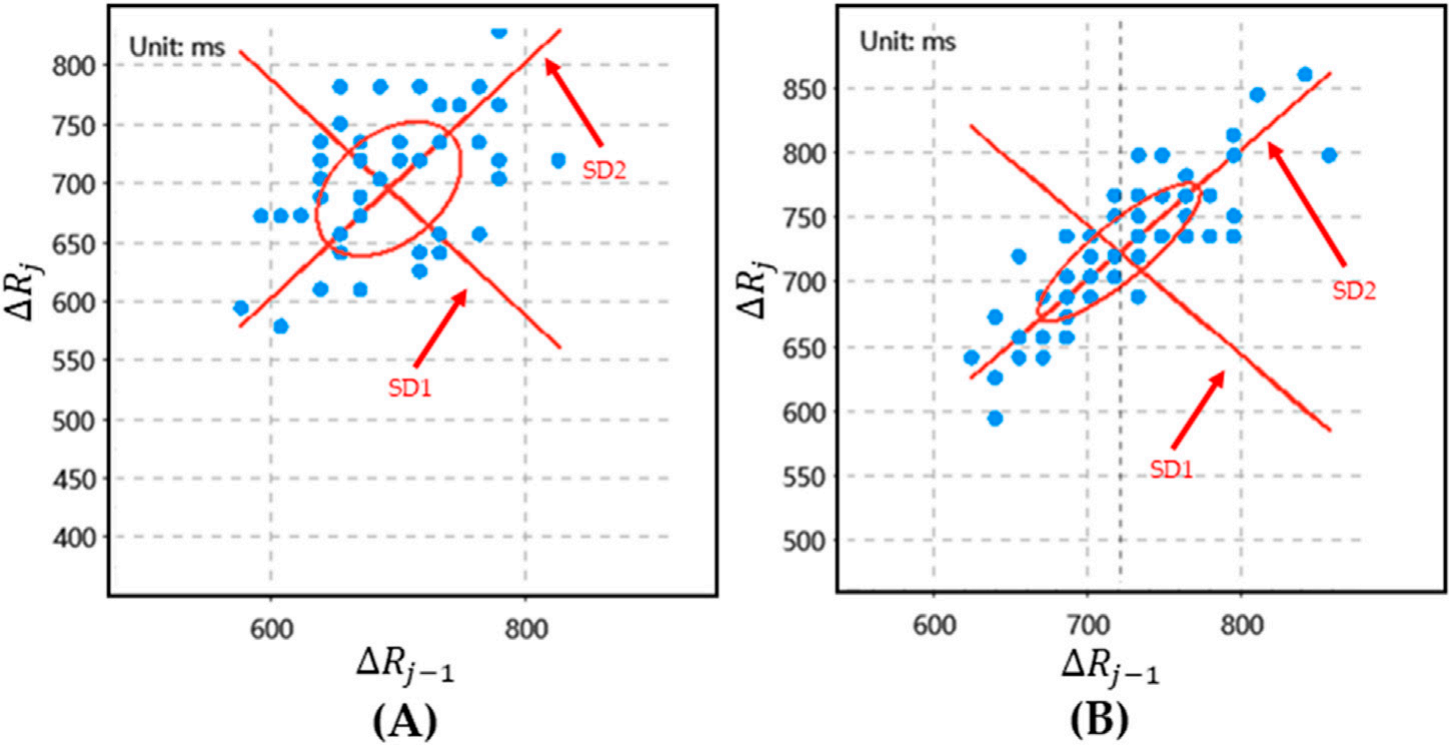
The Poincare plot of HRV. **(A)** The poincare plot of “like” preferences; **(B)** The Poincare plot of “disgust” preference.

By taking the first-order difference of the R-wave time point, all R-R intervals of the HRV signal are obtained and thus the SDNNs are calculated (Yang et al., 2024b). The calculation process of SDNN is as follows (as shown in Equation 1).

$$SDNN=\sqrt{\frac{1}{N-1}\sum_{j=1}^{N}{\left(∆R_{j}-\mu_{∆R}\right)}^{2}}$$ (1)

where *N* is the total number of *R-R* intervals, $∆R_{j}$ is the *j*th *R-R* interval, and $\mu_{∆R}$ is the average of all *R-R* intervals. RMSSD and SDSD can be obtained by second-order differentiation of the R-wave (as shown in Equations 2, 3).

$$\text{RMSSD}=\sqrt{\frac{1}{N-1}\sum_{j=2}^{N}{\left(\Delta R_{j}-\Delta R_{j-1}\right)}^{2}}$$ (2)

$$SDSD=\sqrt{\frac{1}{N-1}\sum_{j=2}^{N}{\left[\left(\Delta R_{j}-\Delta R_{j-1}\right)-\mu \right]}^{2}}$$ (3)

where $\Delta R_{j}-\Delta R_{j-1}$ is the time interval between adjacent *R-R* intervals, μ is the average value of the time interval between adjacent *R-R* intervals. The nonlinear dynamic characteristics of HRV signals can be analyzed through the Poincare plot of HRV signals. Therefore, SD1 and SD2 are calculated (as shown in Equations 4, 5).

$$SD1=\sqrt{\frac{1}{2\left(N-2\right)}\sum_{j=2}^{N}\;{\left[\left(\Delta R_{j}-\Delta R_{j-1}\right)-\mu_{1}\right]}^{2}}$$ (4)

$$SD2=\sqrt{\frac{1}{2\left(N-2\right)}\sum_{j=2}^{N}\;{\left[\left(\Delta R_{j}+\Delta R_{j-1}\right)-\mu_{2}\right]}^{2}}$$ (5)

where $\Delta R_{j}-\Delta R_{j-1}$ is the time interval between adjacent *R-R* intervals, $\mu_{1}$ represents the average value of the time interval between adjacent *R-R* intervals, $\mu_{2}$ is the average of the sum of the time between adjacent *R-R* intervals. Finally, a Poincare plot of HRV signals with different preferences is drawn (as shown in Figure 4).

#### 2.3.2 Frequency domain feature extraction

The frequency domain analysis of physiological signals refers to converting physiological signals into the frequency domain for analysis to understand the frequency components and frequency characteristics in the signals (Chudasama et al., 2022). This study uses Fourier transform and power spectral density analysis methods for frequency domain analysis. For a continuous physiological signal $x\left(t\right)$ , its Fourier transform $X\left(f\right)$ is defined as:

$$X\left(f\right)=\int_{-\infty }^{\infty }\;x\left(t\right)e^{-j2\pi ft}dt$$ (6)

where f is the frequency and j is the imaginary unit. The power spectral density $S_{xx}\left(f\right)$ and the physiological signal $x\left(t\right)$ can be calculated using the autocorrelation function.

$$S_{xx}\left(f\right)=\lim_{T\to \infty }\;\frac{1}{T}{\left|\int_{-T/2}^{T/2}\;x\left(t\right)e^{-j2\pi ft}dt\right|}^{2}$$ (7)

This study focuses on extracting frequency domain features of HRV and RESP signals. For HRV signals, high-frequency (HF) and low-frequency (LF) features are extracted. For RESP signals, the average power frequency is extracted. The discrete versions of Equations 6 and 7 were used.

#### 2.3.3 Feature fusion

Principal Component Analysis (PCA) was employed to reduce the dimensionality of the features. The feature connection to perform feature fusion is utilized. The method of feature connection retains the information of the original features to the greatest extent while combining the characteristics of different physiological signals, providing us with more comprehensive information for subsequent processing and analysis. Specifically, it splices physiological signal features of different types and sources according to feature dimensions to obtain a new feature vector (as shown in Equation 8).

$$X=\left[X_{1},X_{2},X_{3}\right]$$ (8)

where $X_{1}$ represents the features of the first physiological signal, $X_{2}$ represents the features of the second physiological signal, and $X_{3}$ represents the features of the third physiological signal.

### 2.4 Model development

**FIGURE 5:**
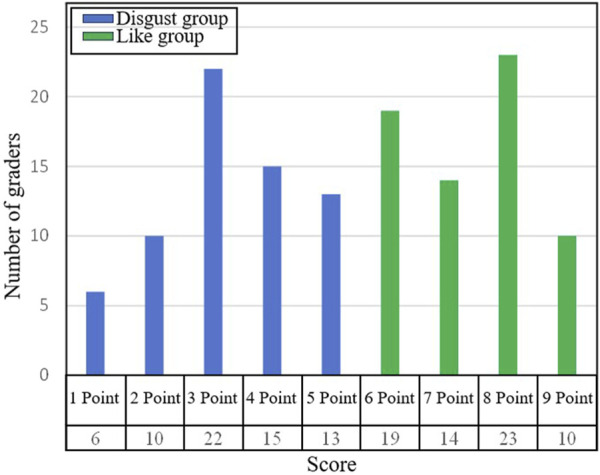
The statistics chart of preference sample. The first row of abscissa is the preference level, and the second row of abscissa is the number of samples under that preference level.

This study divides the nine levels of preferences into two categories, where those with a preference level greater than 5-point are referred to as the ‘like’ group, and those with a preference level less than or equal to 5-point are referred to as the ‘disgust' group (as shown in Figure 5).

The machine learning models we select include Logistic Regression (LR), Support Vector Machine (SVM), Decision Tree (DT), Random Forest (RF), K-Nearest Neighbor (KNN), and Naive Bayes (NB). This paper uses 6-fold cross-validation and evaluate a wide range of machine learning models and hyperparameters.

### 2.5 Model evaluation

This study evaluates the performance of the olfactory preference prediction model, and the model with the highest overall score is set as the final model. 70% of the samples were used as the training set for the model, while 30% were used as the test set. Four indicators of the evaluation metrics are considered, including accuracy, precision, recall, and F1-score. The process of calculating these indicators is as follows (as shown in Equations 9–12):

$$Accuracy=\frac{TP+TN}{TP+TN+FP+FN}$$ (9)

$$Precision=\frac{TP}{TP+FP}$$ (10)

$$Recall=\frac{TP}{TP+FN}$$ (11)

$$F1-score=\frac{2\times TP}{2\times TP+FP+FN}$$ (12)

where *TP* is the number of true positives, *TN* is the number of true negatives, *FP* is the number of false positives, and *FN* is the number of false negatives. The calculation process of the true positive rates *TPR%*, false negative rates *FNR%*, true negative rates *TNR%*, and false positive rates *FPR%* are calculated as follows (as shown in Equations 13–16):

$$TPR\%=\frac{TP}{TP+FN}\times 100\%$$ (13)

$$FNR\%=\frac{FN}{TP+FN}\times 100\%$$ (14)

$$TNR\%=\frac{TN}{TN+FP}\times 100\%$$ (15)

$$FPR\%=\frac{FP}{TN+FP}\times 100\%$$ (16)

Furthermore, this study calculates the confusion matrix of the model and the area under the ROC curve (AUC).

## 3 Results

Using Principal Component Analysis (PCA), the dimensionality of the features was reduced from 15 to 6, resulting in the model achieving optimal accuracy. Under 6-fold cross-validation and hyperparameter tuning, the model did not exhibit significant overfitting. Results of the accuracy, precision, recall, and F1-score of all models are summarized in Table 3. Among them, the decision tree model shows the highest prediction accuracy and achieves the highest scores in precision and F1-score. Although the decision tree model performs worse than the support vector machine model in terms of recall, the gap between them is very small. Therefore, the decision tree model is chosen as the final model after a comprehensive consideration.

**TABLE 3:** The prediction results of six models. The accuracy of the training set is the average value from 6-fold cross-validation.

Model name	Training set	Accuracy	Precision	Recall	F1-score
Logistic Regression	0.88	0.85	0.88	0.78	0.82
Support Vector Machine	0.86	0.82	0.79	0.83	0.81
Decision Tree	0.89	0.88	0.94	0.81	0.87
Random Forest	0.87	0.85	0.88	0.78	0.82
K-Nearest Neighbor	0.83	0.78	0.75	0.71	0.73
Naive Bayes	0.80	0.78	0.78	0.82	0.80

**FIGURE 6:**
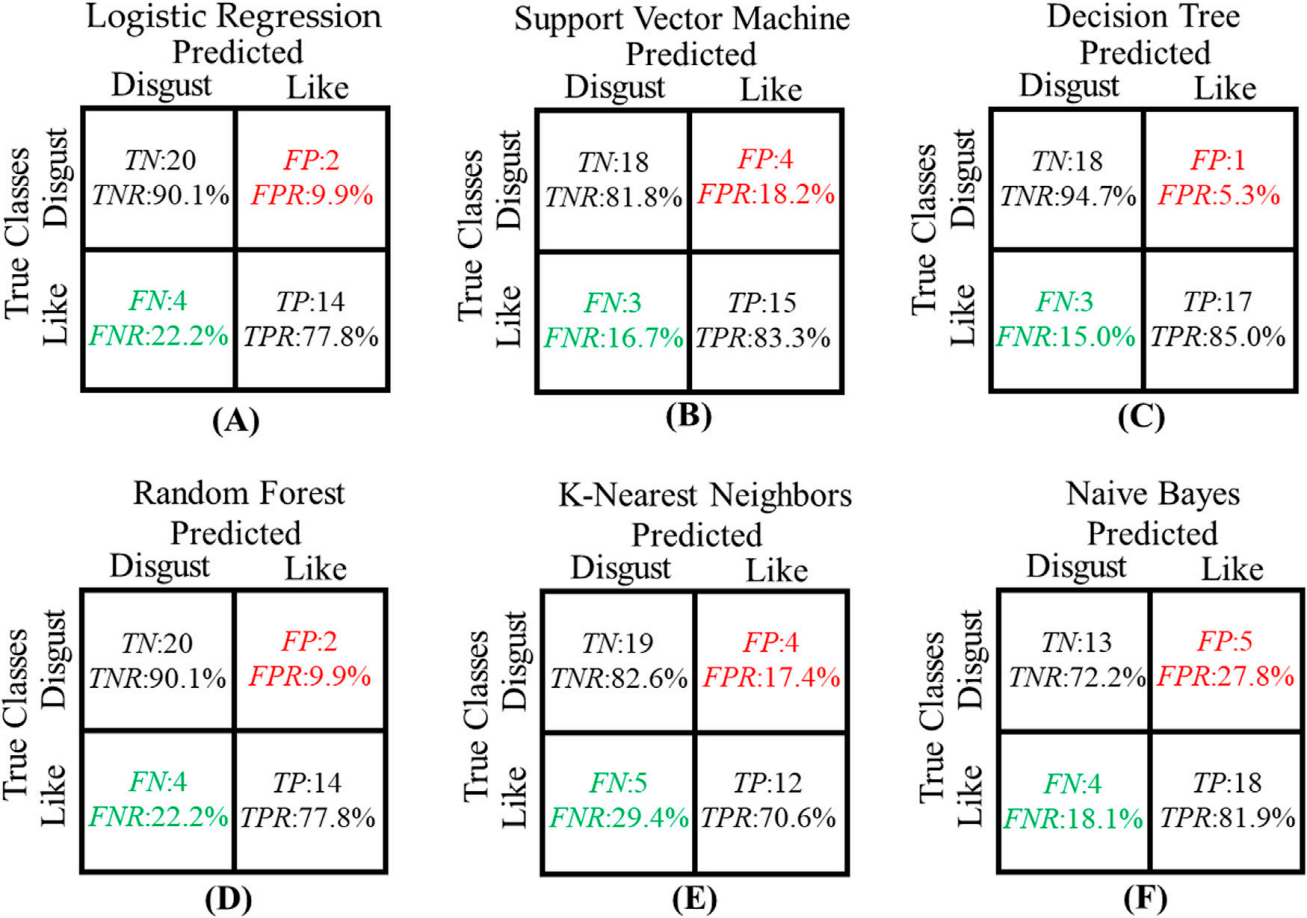
The confusion matrix of six models. **(A)** The confusion matrix of LR; **(B)** The confusion matrix of SVM; **(C)** The confusion matrix of DT; **(D)** The confusion matrix of RF; **(E)** The confusion matrix of KNN; **(F)** The confusion matrix of NB; The red highlighted part is the *FP* and *FPR* of the model. The green highlighted part is the *FN* and *FNR* of the model.

The confusion matrix of all models is shown in Figure 6. The highlighted red part indicates the number of samples that the model incorrectly classifies in the “disgust” class as the “like” class. This is the worst error scenario, meaning that the predicted in-vehicle fragrance preference will increase. Among all models, the Naive Bayes model has the largest proportion of *FP*, reaching 27.8%. In contrast, the decision tree model has the lowest *FP* rate, with only one sample being incorrectly predicted out of 20 “disgust” samples. The highlighted green part indicates the number of samples that the model incorrectly classifies in the “like” class as the “disgust” class. Among them, the KNN model accounts for the largest proportion of *FN*, reaching 29.4%, while the decision tree model still performs the best.

**FIGURE 7:**
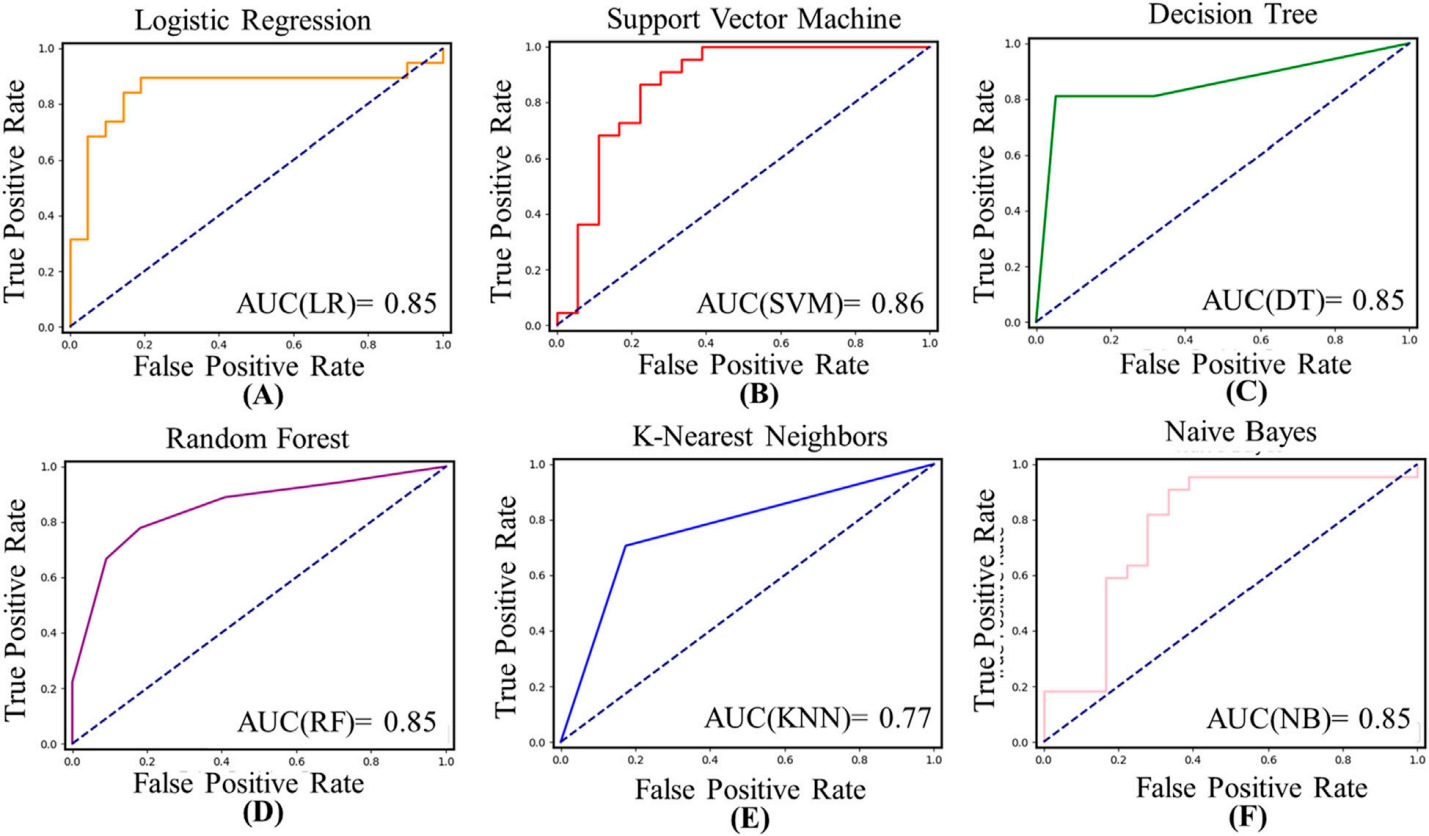
The ROC curves and AUC values of the six models. **(A)** The ROC curves and AUC values of LR; **(B)** The ROC curves and AUC values of SVM; **(C)** The ROC curves and AUC values of DT; **(D)** The ROC curves and AUC values of RF; **(E)** The ROC curves and AUC values of KNN; **(F)** The ROC curves and AUC values of NB.

Figure 7 shows the ROC curve and statistical AUC area of the model. SVM shows the highest AUC value (0.86), while KNN has the lowest AUC value (0.77). However, the observed values show little difference between the several models.

## 4 Discussion

In this study, a machine learning model effectively classifying olfactory preferences based on physiological signals of drivers during in-vehicle fragrance experience is developed. Three physiological signals are adopted as inputs for the model, including heart rate variability, electrodermal activity, and respiratory. In addition, six machine learning models are compared. Finally, the decision tree model is selected as the final model. Therefore, the results of this study are summarized as follows.

### 4.1 Acquisition and processing of physiological signals

It is crucial to consider the physiological state of drivers during driving. In real driving environments, drivers may encounter various situations and pressures, all of which can affect their physiological signals. Through this real driving process, the physiological response of the driver during the experience of in-vehicle fragrance is more closely aligned with the real situation, allowing for more realistic and accurate olfactory preference data to be obtained. Therefore, we set up four experimental stages in the experiment, including the preparation stage, the calm stage, the stimulation stage, and the evaluation stage. Through the driving task in the calm stage, the drivers can maintain a stable state of mind, and their physiological signals show a relatively stable state, with various indicators being controlled within the normal range.

Methods for preference assessment based on physiological signals have been gradually implemented. However, the current lack of standardized evaluation protocols and application guidelines has resulted in assessment outcomes being easily influenced by confounding factors. Ohira and Hirao (2015) selects preferred products by pressing a button, which results in physiological signals being affected. To mitigate the impact of physical movements on physiological signals, a separate stimulation stage was incorporated into our study. Additionally, the implementation of a calm period and baseline processing can effectively mitigate the impact of the placebo effect (Laureanti et al., 2020). However, the duration of the calm period varies across individuals. In this study, the end of the calm period is marked by the participants being fully prepared and the stabilization of their physiological signals, which to some extent reduces the impact of the placebo effect.

**FIGURE 8:**
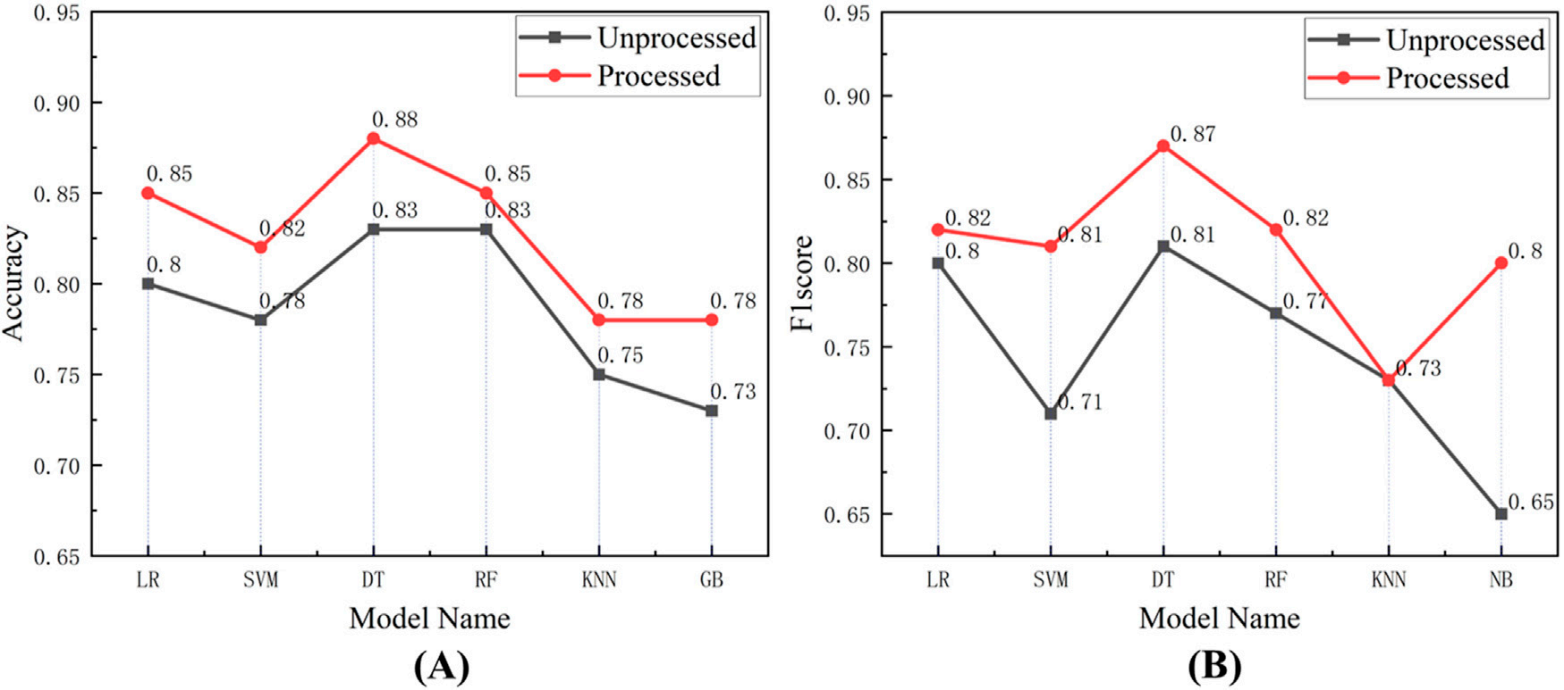
The accuracy and F1-score of six models with two different feature sets as inputs. **(A)** The comparison of accuracy between feature sets with baseline processing and without baseline processing. **(B)** The comparison of F1-score between feature sets with baseline processing and without baseline processing.

In addition, the baseline processing on physiological signal features is performed, which can eliminate the impact of driving. Moreover, the interference of individual physiological differences can be eliminated by calculating the difference between the physiological signal feature values during the stimulation and calm stages. Through the feature difference, the prediction results of the model are more comparable and accurate. Figure 8 shows the accuracy and F1-score of six models with two different feature sets as inputs (the feature sets without and with baseline processing).

As expected, the performance of the model on the dataset with baseline processing increases, and the F1-score also increases. The average accuracy and the average F1-score of the models on the feature set after baseline processing respectively increases by 3.50% and 6.33% compared to the feature set without baseline processing.

The comparison of accuracy rates between single-signal and multi-signal approaches is presented in Table 4. The single-signal models were trained with the same parameter controls as the multi-signal models. The multimodal approach achieved an average accuracy of 82% across the six models, which represents an improvement of 7.7% compared to the HRV signal, 12.7% compared to the RESP signal, and 16.8% compared to the EDA signal. The comparison results indicate that the multimodal approach outperforms the single-signal approach in terms of prediction accuracy.

**TABLE 4:** The comparison of accuracy rates between single-signal and multi-signal.

Input	LO	SVM	DT	RF	KNN	GB
HRV	0.725	0.775	0.825	0.8	0.675	0.7
RESP	0.75	0.725	0.725	0.775	0.625	0.6
EDA	0.675	0.65	0.675	0.75	0.575	0.625
Multimodal	0.85	0.82	0.88	0.85	0.78	0.78

### 4.2 Discussion of prediction results

Artificial intelligence has the potential to significantly enhance the utilization of physiological signals (Autthasan et al., 2023). In this study, a high-accuracy model was developed, enhancing the potential applications of our method. In terms of prediction accuracy, the decision tree model (88%) shows the highest prediction accuracy compared to the other five models (LR: 85%, SVM: 82%, RF: 85%, KNN: 78%, GB: 78%). It is attributed to the following reasons. Firstly, the decision tree models can capture nonlinear relationships between features and can handle features with complex correlations. Therefore, the decision tree models may be more effective than linear models when processing datasets of physiological signals. Secondly, there may be more noise data in the physiological signal dataset due to individual differences and experimental environmental factors. However, decision tree models are insensitive to outliers and can handle imbalanced datasets (Campbell et al., 2022).

Compared to the prediction accuracy, other noteworthy factors are the *TP* and *TPR*. For the application scenario of this research, the model with low *TPR* is closely concerned. The purpose of this study is to help designers design in-vehicle fragrances that meet the olfactory preferences of drivers. The high *TPR* means that in-vehicle fragrances with low preference are used by drivers, which seriously affects their comfort level. In addition, *FN* and *FNR* also have an impact on the practical application of the model. The high *FNR* is disadvantageous for fragrance designers, reduces the preference level of in-vehicle fragrances, challenging to market such products to consumers and resulting in wastage of resources. Setting a classification threshold can effectively reduce *TPR*. However, it should be noted that while reducing *TPR*, *FNR* will increase accordingly. Therefore, it is necessary to adjust the balance between *TP* and *FN* in practical situations to obtain the best classification model.

In addition, an interesting phenomenon is observed. The average *TPR* of the six models is 14.75%, while the average *FNR* is 20.60%. Only the *TPR* of SVM and NB is higher than the *FNR*. This indicates that the performance of the six models in predicting “disgust” samples is better than that of “like” samples. The reason for the above results may be that emotions dominate the olfactory preferences of drivers without considering external factors (such as the brand and color of the fragrance) (Kim et al., 2020; Liu et al., 2021). In the field of emotion research, negative emotions usually have a more significant impact on physiological signals. Bad emotions are accompanied by more pronounced and sustained physiological changes, which are easier to detect and quantify, while the physiological responses to pleasant emotions are more diverse (Domínguez-Jiménez et al., 2020), which can also affect the accuracy of predictions.

### 4.3 Limitations and future work

Some limitations of this study should be acknowledged. Firstly, the age range of the drivers and the sample size in this study may limit the generalizability of the model. However, it is important to note that the current study serves as a preliminary investigation into the field. In future research, collecting data from a more diverse range of drivers to enhance the accuracy and robustness of our prediction model is planned. Secondly, a limited set of physiological signals, including heart rate variability is collected, electrodermal signals, and respiratory signals, to evaluate driver olfactory preferences. While these signals yield promising results, it is essential to consider individual differences and environmental factors to enhance the generalization and stability of the model. In future studies, it aims to incorporate additional peripheral physiological signals to further evaluate driver olfactory preferences and explore the predictive power of different signal combinations. Finally, improving the model is a feasible method to improve the accuracy of prediction. In our future work, the better pattern frameworks and model optimization methods will be explored to improve the prediction accuracy.

## 5 Conclusion

In summary, this study develops a machine learning model that uses the physiological signals (heart rate variability, electrodermal activity, and respiratory signals) of drivers to predict their olfactory preferences. The results of this study have significant practical implications for the design of vehicle comfort, especially for those who are engaged in designing in-vehicle fragrances. Using an olfactory preference prediction model can help manufacturers better understand the needs and preferences of drivers in the early design of in-vehicle fragrances and save more time and costs. In addition, personalized olfactory experiences can be provided for different types of drivers through customized design of in-vehicle fragrances, enhancing the competitiveness and attractiveness of the product. Ultimately, this will widely improve the comfort of drivers during driving.

## Data Availability

The raw data supporting the conclusions of this article will be made available by the authors, without undue reservation.
